# A comprehensive district-level laboratory intervention after the Ebola epidemic in Sierra Leone

**DOI:** 10.4102/ajlm.v8i1.885

**Published:** 2019-10-22

**Authors:** Annelies W. Mesman, Musa Bangura, Sahr M. Kanawa, Joseph S. Gassimu, Kerry L. Dierberg, Mohamed M. Sheku, J. Daniel Orozco, Regan H. Marsh

**Affiliations:** 1 Department of Global Health and Social Medicine, Harvard Medical School, Boston, Massachusetts, United States; 2 Partners In Health, Boston, Massachusetts, United States; 3 Ministry of Health and Sanitation, Koidu, Sierra Leone; 4 Division of Infectious Diseases and Immunology, New York University, New York, New York, United States; 5 Department of Emergency Medicine, Brigham and Women’s Hospital, Boston, Massachusetts, United States

**Keywords:** Sierra Leone, intervention, district hospital, diagnostics, service expansion, mentorship

## Abstract

**Background:**

The 2014–2016 Ebola outbreak exposed the poor laboratory systems in Sierra Leone. Immense needs were recognised across all areas, from facilities, diagnostic capacity, supplies, trained personnel to quality assurance mechanisms.

**Objective:**

We aimed to describe the first year of a comprehensive intervention, which started in 2015, in a public hospital’s general laboratory serving a population of over 500 000 in a rural district.

**Methods:**

The intervention focused on (1) supporting local authorities and healthcare workers in policy implementation and developing procedures to enhance access to services, (2) addressing gaps by investing in infrastructure, supplies, and equipment, (3) development of quality assurance mechanisms via mentorship, bench-side training, and the introduction of quality control and information systems. All work was performed alongside counterparts from the Ministry of Health and Sanitation.

**Results:**

We observed a strong increase in patient visits and inpatient and outpatient testing volumes. Novel techniques and procedures were taken up well by staff, leading to improved and expanded service and safety, laying foundations for further improvements.

**Conclusion:**

This comprehensive approach was successful and the results suggest an increase in trust from patients and healthcare workers.

## Introduction

Laboratories are an often neglected yet crucial part of a functioning health system. Laboratory services are required for clinical diagnosis, patient care, treatment and monitoring and have an important public health role in surveillance of disease and antimicrobial resistance. However, many low-income countries lack adequate laboratory services at the community, district and even national levels, hence laboratories have been referred to as the ‘Achilles heel’ in efforts to combat disease.^1^

The World Health Organization (WHO) has included laboratory services in the ‘basic minimum package’ for health interventions in low-resource settings,^2^ and it is recommended to invest in integrated laboratory services, rather than disease-specific programmes.^3^ However, despite these recommendations, laboratories receive little attention in most health intervention programmes.^4^ Laboratory capacity building is often restricted to short training or equipment donations (many times, equipment is obsolete or being phased out of facilities in high-income countries).^5,6^ Health professionals in low-resource settings are familiar with the sight of unused or broken machines scattered throughout hospital grounds.^5,6^ Because a trained workforce and quality assurance mechanisms, such as the routine maintenance of equipment, are often lacking, patients and clinicians often mistrust diagnostic services.^4,7,8,9,10^

In Sierra Leone most people live below the poverty line, life expectancy and adult literacy in 2016 were as low as 51.3 years and 48.1% respectively while the maternal mortality rate of 1360 per 100 000 live births – was among the highest in the world.^11^ The national public health system has been devastated by a series of events including an 11-year civil war from 1991 to 2002, and the 2014–2016 Ebola virus disease (EVD) outbreak, during which many health workers lost their lives.^12,13^ The Ebola outbreak exposed a fragile health system, with weak diagnostic networks in the region. Public laboratories were not equipped with facilities for EVD diagnostics or adequate sample referral systems, leading to long delays in care, which contributed to higher mortality for both non-EVD and EVD patients.^14,15,16^ This situation changed only when international organisations and governments offered temporary, deployable laboratory support.

In June–July 2015, after the peak of EVD transmission, the Sierra Leone Ministry of Health and Sanitation (MOHS) conducted a comprehensive evaluation of the national laboratory system, using an adapted WHO laboratory assessment tool,^17,18^ which was funded by the United Kingdom Department for International Development.

Three hundred and fifteen (315) laboratories, ranging from community health centre laboratories to all district and regional hospital level laboratories, were assessed. The study identified gaps in systems, supplies, infrastructure and quality, including insufficient numbers of adequately trained personnel to respond to outbreaks. Of 20 public hospitals, 11 (55%) had no reliable water supply, 16 (80%) lacked 24-h electricity, and five (25%) had no freezer space. Regarding testing resources, 16 (80%) offered no blood biochemistry tests, and only three (15%) had electrolytes and liver function tests. Standard operating procedures for available tests were absent at 16 (80%) sites. The majority (83%) of the facilities had no guidelines for specimen referral. Additionally, while acknowledging the needs, the report stressed limited government resources available to invest in laboratory services.^18^

At the invitation of the Sierra Leonean government, the non-profit organisation Partners In Health (PIH) started working in Sierra Leone during the Ebola outbreak in 2014. By 2015, PIH shifted its focus from emergency response and committed to providing long-term comprehensive support at the Koidu Government Hospital (KGH), the district hospital in Kono. The intervention in the district laboratory was initiated in October 2015, a few months after the MOHS laboratory assessment. Here, we describe the first year of this programme and summarise initial outcomes, as a replicable model for laboratory strengthening in low-resource settings.

## Intervention

### Ethical considerations

No identifiable patient data were collected for this study.

### Baseline setting

Kono is a district in eastern Sierra Leone (506 867 population). Rich in diamond mines, Kono was the epicentre of the civil war. There were 301 confirmed EVD infections in the district.^19^ In October 2015, KGH, serving the entire district population, had unreliable water and electricity, insufficient numbers of trained staff, and was underused by the community. In the hospital laboratory, electronic equipment and computers were not used. Supplies of diagnostic tests, consumables, and reagents from the national level were unreliable. Standard operating procedures were largely absent, unknown, or poorly implemented, leading to unsafe situations for staff and patients. Patient fees were applied (at times inconsistently) per individual test.

The KGH laboratory was staffed by a total of 15 technicians and technician assistants, of whom several were not paid or had no laboratory training. This baseline capacity was reflected in the 2015 MOHS national laboratory assessment, in which the overall score for the laboratory, based on availability of supplies, equipment, and quality assurance was 30%, ranking KGH among the five lowest scoring districts.^18^

During the Ebola outbreak, patients with suspected EVD were quarantined in a holding unit on the hospital grounds. After receiving a positive or negative test result, they were referred to an Ebola treatment centre or the KGH wards. Koidu Government Hospital laboratory staff collected blood samples from patients in the holding unit, and received oral swabs from deceased individuals in the district. Referral of all specimens to a Public Health England-supported laboratory in the region was well organised and strictly regulated. In contrast, referral systems for other samples (i.e. for testing or surveillance of other diseases) was poor. External quality assurance mechanisms, such as reading tuberculosis microscopy slides at the national level, were hampered by the outbreak, and for biosafety reasons referral of sputum samples for culture or drug sensitivity testing at the supranational laboratory was on hold.

### Program objective and strategy

Partners In Health worked alongside local MOHS counterparts to support their laboratory strategy and accompany them in the improvement process. Partners In Health’s health system strengthening model – also referred to as ‘stuff, staff, space, and systems’ – incorporates investments in materials, human resources, facilities, and systems.^20^ This comprehensive approach was applied to our laboratory programme, aiming to improve quality from pre- to post-analytical stages of diagnostics, while meeting national strategies and offering the services described in MOHS policies.^15,18^ In the first year of the intervention, the programme focused on the most crucial or attainable issues (listed below), while laying a foundation for continuous and structural improvements.

### Supporting local health authorities on policy implementation to enhance access to services

- The laboratory diagnostic menu was revised to align with Sierra Leone’s national guidelines for district level services and WHO policies (Table 1). New systems and tests were introduced gradually throughout the year to ensure proper time for training and adjustment to novel modalities for both laboratory staff and clinicians.
- To improve access to services for patients, a flat laboratory fee (5000.00 SLL [Sierra Leonean Leone] or $0.70 [United States dollars]) for outpatients was introduced. Inpatient laboratory services were free of charge.
- In collaboration with the hospital’s HIV and tuberculosis clinics, referral systems were revised to (1) ensure proper and rapid transport of specimens from community clinics to the laboratory for diagnostic testing and treatment monitoring and (2) refer patients with positive test results from the laboratory to the HIV or tuberculosis clinic for further testing and counselling. As the hospital laboratory relied on smear microscopy for tuberculosis diagnosis, a referral system with a nearby PIH-supported outpatient clinic was set up for molecular testing for tuberculosis for patients suspected to have multidrug-resistant or extrapulmonary tuberculosis.
- Community Health Workers were given a central role in HIV and tuberculosis referral processes. All Community Health Workers at KGH were trained in HIV and tuberculosis care and equipped to guide patients through the hospital system, accompanying them to and from the laboratory. Additionally, Community Health Workers coordinated collection and transport of sputum samples for tuberculosis testing from patients in the community to the laboratory.
- To improve inpatient care, routine blood draws were introduced in all wards in the mornings, and the results were disseminated in the afternoon.

**TABLE 1:** Diagnostic testing menu for Koidu Government Hospital central laboratory, Sierra Leone, 2016.

Test	Technology
Hb/Hct	Hb POCT/Haematology analyser
Complete blood count	Haematology analyser
Sickling	Sodium metabisulphate
Malaria	Smear/RDT
Urinalysis	10P strips; microscopy
Stool examination	Microscopy
Microbiology	Gram staining; microscopy
Glucose	POCT Glucometer
Typhoid	IgM RDT/WIDAL
HBV, HCV	HBsAg RDT, HCV Ab RDT
Chemistries (RFT, LFT, lipids, electrolytes)	Semi-automated analyser/POCT device
HIV test	HIV 1/2 Ab RDT
CD4 monitoring	CD4 analyser
Cryptococci	Ag RDT
Tuberculosis (AFB)	Smear microscopy
Tuberculosis (molecular test)	Referral
Pregnancy	HCG strip
Syphilis	Rapid Plasma Reagin
Blood grouping	Tile method†

Hb, haemoglobin; Hct, haematocrit; POCT, point-of-care-test; RDT, rapid diagnostic test; 10P, 10 parameter; IgM, immunoglobulin M; LFT, liver function test; RFT, renal function test; AFB, acid fast bacilli; Ab, antibody; CD4, cluster of differentiation 4; HBV, hepatitis B virus; HCV, hepatitis C virus; HCG, human chorionic gonadotropin; Ag, antigen.

† , performed at blood bank, not included in the scope of this report.

### Addressing gaps in the system

- *Infrastructure*: Utility improvements were made at the hospital, including the laboratory, ensuring water and electricity were available 24 hours per day seven days per week. Minor infrastructure and repair work was completed at the laboratory building. The laboratory areas were reorganised, and a separate space for phlebotomy and sample collection was built to improve both patient and sample flow, as well as safety and privacy. Air conditioning was installed to protect equipment, as well as fans (to increase air circulation) and UV lights, two recommended interventions to reduce the risk of tuberculosis transmission.
- *Procurement and supplies*: A procurement and cold-chain supply system was established to fill gaps and support transport of MOHS supplies. Procurement was organised in biannual international orders and quarterly regional and local orders to ensure uninterrupted availability of consumables and reagents and to reduce stock-outs and expiration. Refrigerators and freezers were bought and installed to expand cold storage capacity in the laboratory, hospital and district warehouses. Replenishment of supplies to the laboratory from a central warehouse took place on a weekly basis.
- *Equipment*: To improve quality and expand diagnostic services, novel equipment was introduced. Technicians from respective companies installed and provided training on larger equipment. Smaller equipment was introduced by the laboratory manager:
  ■ Electronic light-emitting diode (LED) microscopes for tuberculosis and malaria diagnosis, as well as urine and stool microbiological diagnostics
  ■ Electrical centrifuges for separation of plasma and serum and sedimentation of urine to replace hand centrifuges
  ■ Haematology analyser (20 parameter) for complete blood counts
  ■ Biochemistry analyser, semi-automated
  ■ Point-of-care testing equipment for electrolytes, haemoglobin and blood glucose.

### Supporting operations and improving quality assurance

- *Management and education*. Partners In Health recruited a laboratory manager, who worked with the MOHS laboratory superintendent on supervision and management. Provision of ongoing mentorship and training to improve test performance were essential parts of the programme. In addition to didactic training for the entire team, individual technicians received bench-side training to use novel equipment during multiple weeks of mentorship.
- *Quality control and assurance*:
  ■ For every new test and procedure, a standard operating procedure was developed to standardise the quality of testing and biosafety.
  ■ Daily, weekly and monthly controls, maintenance and calibration were implemented for all novel equipment according to manufacturers’ guidelines; contracts and service agreements were obtained for all relevant equipment to cover regional or international technical support services and warranty.
  ■ Daily temperature monitoring was introduced for all cold storage space in the laboratory and warehouses.
- *Data management and information systems*:
  ■ To improve documentation and registration, forms when needed were updated, and standardised registration for all requests and results was introduced in the laboratory.
  ■ The laboratory received monitoring and evaluation support to develop data collection tools and continuously monitor operations, including inventory and consumption rates.
  ■ By ensuring provision of test and consumption data to national programmes, including the National AIDS Secretariat and National AIDS Control Programme, National Tuberculosis Control Programme, and the Malaria Control Programme, sufficient HIV, tuberculosis and malaria testing materials were supplied from national stores for these Global Fund-supported programmes. Partners In Health filled any remaining supply gaps.

## Outcomes

### Increased patient and testing volumes

**FIGURE 1:**
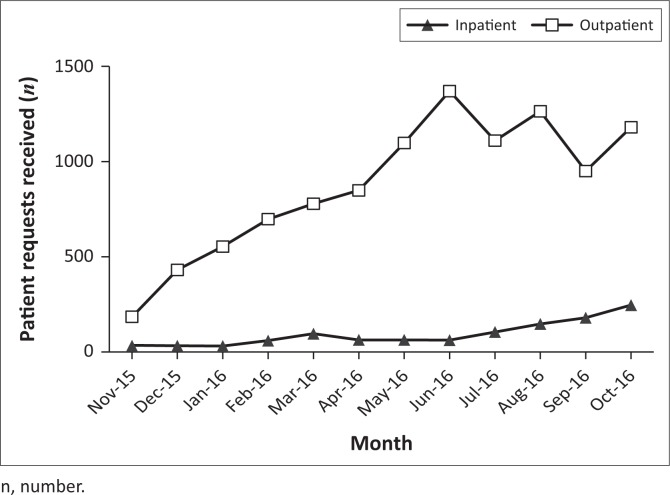
Patient request volume by month, Koidu Government Hospital central laboratory, Sierra Leone, November 2015–2016. Individual patient test requests per month separated for inpatient (black triangles) and outpatient (open squares) departments.

Between November 2015 and October 2016, the volume of individual outpatient test requests increased 5.8 times over baseline, from 246 to 1428 per month (Figure 1). During this timeframe, PIH’s health system programming primarily supported inpatient and outpatient HIV and tuberculosis care, but was not involved in general outpatient services. Therefore, this increase in outpatient visits (including from surrounding districts) suggests increased trust in the health system and KGH among national staff and local community members, as evidenced by increasing test requests and larger patient volumes, and potentially reflects expanded service delivery.

**FIGURE 2:**
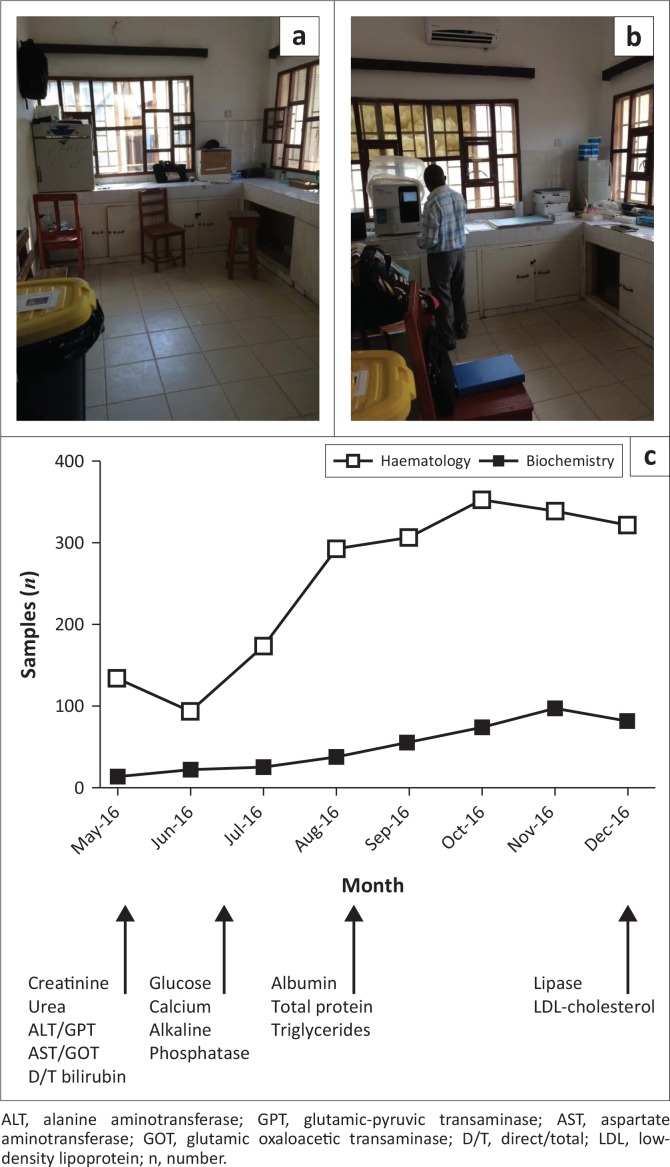
Introduction of haematology and biochemistry services, Koidu Government Hospital central laboratory, Sierra Leone, 2016. (a) The haematology laboratory before introduction of equipment, with a broken incubator for storage (left) and (b) after installation of air conditioning and equipment, with a technician turning on the haematology machine (right). (c) Number of haematology (closed squares) and biochemistry (open squares) samples received for testing during the first six months of introducing the new equipment. Below the x-axis is indicated when various biochemistry tests were introduced.

After improving infrastructure and preparing the laboratory and technicians for new equipment, semi-automated haematology and biochemistry analysers were installed in May 2016. During the first six months after introduction, this new equipment enabled the laboratory to perform a total of 224 liver and renal function tests for HIV and tuberculosis patient monitoring and inpatient care, as well as 1352 complete blood counts for inpatient and outpatient care (Figure 2). Overall, the programme and, specifically, the introduction of new machines boosted staff morale. Introducing new procedures and tests required a moderate amount of initial cost and training, but was absorbed relatively easily by the national MOHS laboratory team.

**FIGURE 3:**
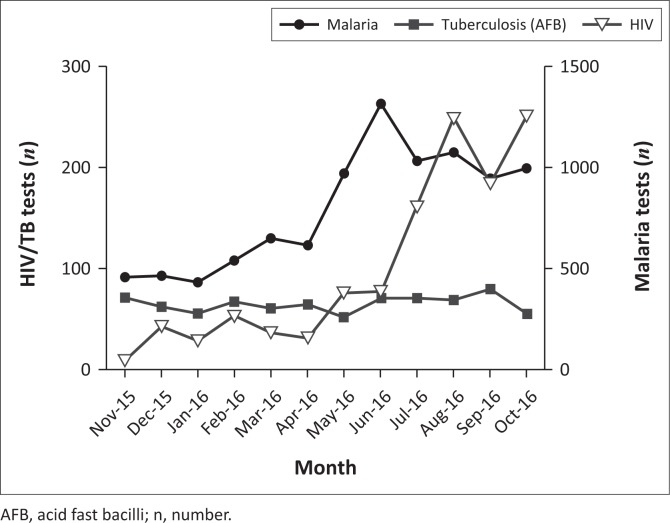
Testing volumes of HIV, tuberculosis and malaria by month, Koidu Government Hospital central laboratory, Sierra Leone, November 2015–2016. Number of tests performed per month for HIV (open triangles, left y-axis), tuberculosis (AFB, closed squares, left y-axis) and malaria (closed circles, on right y-axis).

To illustrate the effect of our intervention on diagnostics of HIV, tuberculosis and malaria – which are all national and WHO priorities – testing volumes are presented in Figure 3.These numbers reflect volumes within the laboratory and exclude point-of-care HIV and malaria tests performed by clinicians and support staff in the wards and HIV clinic.

- *HIV*: Prior to the intervention, almost all HIV tests for outpatients were performed at the HIV clinic on the hospital grounds and on the wards for inpatients. The laboratory performed only eight HIV tests in November 2015; this number increased to 251 in October a year later. This result was achieved by training technicians in testing procedures, patient confidentiality, and improving communication with clinicians, HIV clinic staff, and Community Health Workers. This change gave the HIV clinic staff more time to focus on other tasks, and increased access to testing services for outpatients, without a required visit to the HIV clinic.
- *Tuberculosis*: Repair and ongoing maintenance of a *Mycobacterium tuberculosis* molecular testing platform and supply of cartridges located at our partner outpatient health clinic enabled the testing of 66 specimens (for patients suspected of having multidrug-resistant tuberculosis or extrapulmonary tuberculosis) during the first six months of this system.
- *Malaria*: Throughout the year, the laboratory tested 9501 patients for malaria, with a peak in volume (and positivity rate, data not shown) in the rainy season from May to September. To support the increase in volume, PIH supplied rapid diagnostic tests in addition to slide tests to reduce the burden on staff.

## Discussion

As a result of this programme, the diagnostic capacity at KGH was expanded and improved and an increase in patient visits was recorded. This suggests that an improvement in environment, organisation and access to diagnostics allowed for more trust and satisfaction with the system by patients and clinicians, as has been shown by others.^8,9^

This programme had several limitations. All operational and clinical support was part of a hospital-wide health-strengthening programme. These combined efforts had the immeasurable effect of improving service delivery and trust among patients, community and hospital staff, likely contributing to the increase in laboratory utilisation as well. Therefore, we cannot observe our outcomes in isolation from other interventions. Secondly, our data demonstrate improved access to laboratory services, as more patients visited the laboratory, but do not quantify improved quality or how access to these services changed clinical practice and outcomes. A laboratory satisfaction survey would provide better information about perceived laboratory services.

In a MOHS 2016 progress report (personal communication), the laboratory score increased from 65% to 87% for availability of tests, supplies, and standard operating procedures. This indicates that improvement of laboratory services was recognised by the government. Unfortunately, this report was not published nationally and the structure differed from the 2015 comprehensive assessment and hence cannot be compared with other districts or the 30% score in the previous report. Lastly, this score is not synonymous with quality services; for example, the KGH laboratory lost credits in 2016 after replacing the haemoglobin colour scale with an automated analyser, because only the colour scale was provided by MOHS and included in the assessment.

It is the mission of PIH to strengthen public health systems to ensure the access of health services to people living in resource-limited settings. Investing in public sector laboratory services though a comprehensive approach has proven effective in other countries.^21^ In rural Haiti and Rwanda, multidrug-resistant tuberculosis and pathology departments are in operation or being built. These established ‘beacon facilities’ serve the poorest populations, function as teaching sites, and are considered a model for health service provision on national and regional levels.^20^ While partnering with governments is central to rebuilding public health infrastructure, it may also limit certain programmatic decisions. For example, PIH aims to offer free health care for all, but the Sierra Leone public system and many other health services in low-resource countries are based on cost recovery. We could therefore only lower the costs per patient for laboratory services, not eliminate these costs for all patients. Although our staff would not deny anyone care, and some patients received free care according to national policy (those under the age of 5 years, pregnant and lactating women, EVD survivors, and patients receiving HIV or tuberculosis care), over 50% of the population lives on less than $2.00 per day^11^ and these fees may still pose a barrier to many for accessing health services.

To remove these barriers and rebuild health systems, advocacy and collaboration with national programmes are needed. These efforts are also required regarding testing policies. There are discrepancies between national guidelines, WHO recommendations, and accepted standards for tests used in high-income countries. For many low-income countries, cheap testing methods for diseases such as HIV and tuberculosis are considered acceptable^22^ and on many occasions point-of-care tests are used to replace instead of complement more accurate or sensitive tests.^4^ More sensitive procedures, such as nucleic acid amplification tests and microbiological culture, in Sierra Leone and many other countries, are limited to reference laboratories, which cannot serve the total population. The lack of logistics, supply system, referral networks and facilities are considered major bottlenecks to introduce novel diagnostics,^23^ creating a vicious cycle and preventing the introduction of new technologies. Very recently, following calls from experts,^24^ WHO developed the first version of an ‘essential diagnostics list’^25^ – 40 years after the release of the ‘essential medicines list’, which has been updated every 2 years since 1977. Furthermore, WHO, the United States Centers for Disease Control and Prevention and the Association of Public Health Laboratories developed a Global Laboratory Leadership Programme.^26^ All of these steps may contribute to a new commitment for better quality testing globally.

The intervention described here is still ongoing as PIH continues to support the MOHS staff in the laboratory and other clinical service delivery. To improve the quality of diagnostic services, PIH has recruited a quality officer to support the laboratory manager. The next steps of the programme include, but are not limited to, enrolment of tests into (national) external quality assurance systems, strengthening the referral system, introduction of microbiological culture assays and equipment for automated tuberculosis and HIV viral load detection, eventually leading to improved quality services, emergency preparedness, and first steps towards accreditation.^15^ In many ways, KGH is representative of an extremely underserved district hospital and we offer this example of our model to demonstrate how services can be reliably and sustainably strengthened in similar contexts.

Lessons learned

- A successful comprehensive laboratory intervention includes long-term mentorships and involves addressing policies, infrastructure and quality assurance.
- Strengthening the laboratory system requires the active involvement of stakeholders, including clinicians, HIV and tuberculosis services, hospital staff, and Community Health Workers.
